# Induction or Adjuvant Chemotherapy in Patients with Locally Advanced Nasopharyngeal Cancer Receiving Chemoradiotherapy? A Turkish Oncology Group Study

**DOI:** 10.3390/jcm14124189

**Published:** 2025-06-12

**Authors:** Serhat Sekmek, Aysel Oguz, Melek Karakurt Eryilmaz, Murat Araz, Sedat Biter, Mehmet Mutlu Kıdı, Ertugrul Bayram, Efe Cem Erdat, Arzu Yasar, Rumeysa Colak, Mesut Yilmaz, Gizem Bakir Kahveci, Didem Divriklioglu, Elvin Chalabiyev, Sercan Aksoy, Sema Nur Ozsan Celebi, Hakan Kosku, Mesut Yılmaz, Ilhan Hacibekiroglu, Haydar Temizyurek, Kubra Canaslan, Gorkem Turhan, Ahmet Kadıoglu, Seda Jeral, Mehmetcan Atak, Huseyin Atacan, Anil Ozbay, Tugay Atasever, Mustafa Seyyar, Pervin Can Sanci, Bahadir Koylu, Nargiz Majidova, Erkan Arpaci, Muhammed Bulent Akinci, Dogan Uncu, Gokhan Ucar

**Affiliations:** 1 Department of Medical Oncology, Ankara Bilkent City Hospital, 06800 Ankara, Turkey; ozsan.semanr@gmail.com (S.N.O.C.); hakankosku@gmail.com (H.K.); mbakinci@gmail.com (M.B.A.); doganuncu@yahoo.com (D.U.); gokhanucar_1@hotmail.com (G.U.); 2 Department of Medical Oncology, Necmettin Erbakan Unıversıty, 42090 Konya, Turkey; drayseloguz@gmail.com (A.O.); drangelkarakurt@hotmail.com (M.K.E.); zaratarum@yahoo.com (M.A.); 3 Department of Medical Oncology, Cukurova University, 01790 Adana, Turkey; sedatb23@hotmail.com (S.B.); mehmetmutlu_01@hotmail.com (M.M.K.); ertugrulbayram84@gmail.com (E.B.); 4 Department of Medical Oncology, Ankara University, 06230 Ankara, Turkey; cemerdat@gmail.com (E.C.E.); arzuyasar@gmail.com (A.Y.); 5 Department of Medical Oncology, Bakırköy Dr. Sadi Konuk Training and Research Hospital, 34147 Istanbul, Turkey; colak.rmys@gmail.com (R.C.); mesutyilmaz12@yahoo.com (M.Y.); 6 Department of Medical Oncology, Trakya University, 22030 Edirne, Turkey; bakirkahvecigizem@gmail.com (G.B.K.); dr_didemeroglu@hotmail.com (D.D.); 7 Department of Medical Oncology, Hacettepe University, 06100 Ankara, Turkey; elvin.chelebi@gmail.com (E.C.); saksoy07@yahoo.com (S.A.); 8 Department of Medical Oncology, Sakarya Training and Research Hospital, 54100 Sakarya, Turkey; mstsnm08@gmail.com (M.Y.); ilhanhbo@hotmail.com (I.H.); 9 Department of Medical Oncology, Afyonkarahisar Health Sciences University, 03030 Afyon, Turkey; temizyurek.71@hotmail.com; 10 Department of Medical Oncology, Dokuz Eylul University, 35390 Izmır, Turkey; 11 Department of Medical Oncology, Karadeniz Technıcal University, 61080 Trabzon, Turkey; gorkemturhan@gmail.com; 12 Department of Medical Oncology, UHS Dr Abdurrahman Yurtaslan Ankara Oncology Training and Research Hospital, 06200 Ankara, Turkey; ahmetkadiogluu@gmail.com; 13 Department of Medical Oncology, Cerrahpasa School of Medicine, 34098 Istanbul, Turkey; sedajeral@gmail.com; 14 Department of Medical Oncology, Ankara Etlik City Hospital, 06170 Ankara, Turkey; mehmetcanatakk@gmail.com; 15 Department of Medical Oncology, Gülhane Training and Research Hospital, University of Health Science, 06010 Ankara, Turkey; drhuseyinatacan@gmail.com; 16 Department of Medical Oncology, Kocaeli City Hospital, 41060 Kocaeli, Turkey; anilozbay@hotmail.com; 17 Department of Medical Oncology, Prof. Dr. Cemil Tascıoglu City Hospital, 34384 Istanbul, Turkey; tugay.atasever@hotmail.com; 18 Department of Medical Oncology, Gaziantep City Hospital, 27470 Gaziantep, Turkey; mustafaseyyar27@hotmail.com; 19 Department of Medical Oncology, Kocaeli University, 41380 Kocaeli, Turkey; ppervincan@hotmail.com; 20 Department of Medical Oncology, Koc University, 34450 Istanbul, Turkey; bahadirkoylu@gmail.com; 21 Department of Medical Oncology, Marmara University, 34854 Istanbul, Turkey; nergiz.mecidova1991@gmail.com; 22 Department of Medical Oncology, Sakarya Adatip Hospital, 54050 Sakarya, Turkey; doktorerkan2001@yahoo.com

**Keywords:** nasopharyngeal cancer, induction, adjuvant, chemotherapy

## Abstract

**Background/Objectives:** In this retrospective study, we aimed to compare the efficacy, survival, and toxicity results of induction (IC) or adjuvant (AC) treatment with chemoradiotherapy (CRT) in locally advanced nasopharyngeal cancer (NPC). **Methods:** A total of 405 patients from 22 different centres in Turkey, belonging to the Turkish Oncology Group (TOG), was included. The primary endpoints were overall survival (OS) and progression-free survival (PFS), and the secondary endpoints were safety and toxicity. **Results**: The median age of the patients included in the study was 49 (18.2–91.5) years. In total, 298 (73.6%) of the patients were male. Of the 405 patients, 258 (63.7%) received IC and 147 (36.3%) received AC treatment. When OS and PFS analyses were performed in terms of age, gender, T and N stages, pathological features, and treatments received, no effect of any variable on prognosis was observed. For the overall group, the median estimated OS was 137.3 months (the Kaplan–Meier statistical method could not reach the 95% confidence interval [CI]). For the IC group, the median estimated survival was 137.3 months (95% CI: 111.4–163.3), whereas the Kaplan–Meier statistical method could not estimate survival for the AC group. No statistically significant difference was observed between IC and AC groups in terms of OS (*p* = 0.209) or PFS (*p* = 0.248). Grade 3–4 side effects were observed in 12% of patients in the IC group and 29.9% of patients in the AC group. Treatment was discontinued due to toxicity in 5 patients (1.9%) in the IC group and 18 patients (12.2%) in the AC group. **Conclusion:** No difference in OS or PFS was observed between AC and IC treatments. More grade 3–4 side effects were observed in the AC-treated group and early discontinuation rate was higher.

## 1. Introduction

Nasopharyngeal cancer (NPC) is a malignant tumour arising from the nasopharyngeal epithelium and usually originating from the pharyngeal recess [1]. According to 2020 data, there are 133,000 new cases and 80,000 deaths per year in the world [2]. It differs from other head and neck cancers with its epidemiological, pathological aspects, and treatment modalities [3]. While the main treatment method in other head and neck cancers is surgery, the primary treatment method in NPC is radiotherapy (RT) [4].

Since NPC is a highly sensitive tumour to ionising radiation, RT alone is sufficient in early-stage disease (T1–2, N0) [5]. However, in locally advanced disease, administration of chemotherapy together with radiotherapy (CRT) has been shown to increase survival [6]. In concurrent chemotherapy (CT), cisplatin treatment is usually applied [7]. CRT is applied instead of RT in high-risk T1–2 and N1 patients and T3, N0 patients. After the demonstration of the efficacy of CRT, adjuvant chemotherapy (AC) after CRT has been investigated. There are studies showing that CRT plus AC (CRT-AC) contributes more to survival than CRT alone, especially in T3, N1, T4, and N2–3 patients [8,9].

The fact that the CT tolerance of patients receiving AC after CRT is very low and that the results do not show the expected survival benefit due to the inability of patients to receive their treatment effectively has led to induction CT (IC) studies in which patients receive CT before CRT [10]. Similarly to adjuvant treatment, better survival results were obtained with CRT after IC (IC-CRT) in T3 and N1, as well as T4 or N2–3 patients compared to patients receiving CRT alone [11]. Since the survival contributions of both AC and IC are similar and no clear superiority of one treatment modality over the other has been demonstrated to date, the treatment modality preferred in various clinics is also different.

There are very few studies in the literature comparing induction and adjuvant treatments [12]. In these studies, one-to-one comparisons were generally not made between these treatment modalities. Instead, meta-analyses were used for comparison or comparisons were made between several subgroups within a trial. In the studies performed so far, there are results showing that either treatment is more effective than the other [13,14]. In this retrospective study, we aimed to compare the efficacy, survival, and toxicity results of IC-CRT and CRT-AC treatments in locally advanced NPC.

## 2. Methods

This retrospective study included 405 patients from 22 different centres in Turkey diagnosed between 2007 and 2024. Patients over 18 years of age, pathologically diagnosed with NPC, in locally advanced stage, and receiving IC-CRT or CRT-AC treatment were included in the study. In the centres, careful attention was given to the inclusion of stage three or four patients in the IC or AC group, and patients with lower stages were excluded from the study. The patients were assigned to receive either AC or IC treatment, as decided by the Multidisciplinary Tumour Boards at their treatment centre. The CT received by the patients during AC or IC treatments and during CRT were determined according to the physician’s choice. Patients under 18 years of age, without a diagnosis of NPC, with early-stage disease (T1–2, N0), metastatic disease, receiving only CT, only RT, or only CRT were excluded from the study. Patients’ characteristics, treatments, pathological features, laboratory results, and disease progression were retrospectively screened from their medical files.

The primary endpoints were overall survival (OS) and progression-free survival (PFS). OS was defined as the time from the date of diagnosis until death from any cause. PFS was defined as the time from the date of initial diagnosis until progression or death from any cause. The secondary endpoints were safety and side effects of treatment. The patients underwent imaging with the radiological method chosen by their physicians at most every 3 months from the beginning of their treatment. The results of radiological imaging methods were recorded. The treatment-related side effects of the patients were retrospectively reviewed from the patient files. The side effects occurring during the treatments were recorded using Common Terminology Criteria for Adverse Events (CTCAE) version 5.

IBM SPSS version 25 was used for all statistical analyses. Histograms and the Shapiro–Wilk test were used to detect normal distribution. Normally distributed continuous variables were described as mean ± standard deviation, while non-normally distributed variables were described as median (min–max). Two group comparisons of continuous variables were made by the Mann–Whitney U test. The chi-squared or Fisher exact test was used for categorical comparisons. Kaplan–Meier survival curves and Cox regression analysis were used for survival and prognostic factors. A *p*-value < 0.05 was considered significant.

## 3. Results

The median age of the patients included in the study was 49 (18.2–91.5) years. In total, 298 (73.6%) of the patients were male. Of the 405 patients, 258 (63.7%) received IC and 147 (36.3%) received AC treatment. Baseline clinicopathological characteristics of the patients are shown in Table 1. When the age, gender, T and N stages, pathological features, and chemotherapies received during CRT were compared between the AC and IC groups, only Epstein–Barr virus (EBV)-encoded small RNAs (EBER) positivity was observed between the two groups. No statistical difference was observed between the two groups in terms of other variables (Table 2).

When the treatments received by the patients together with CRT were evaluated, the most commonly administered chemotherapies in the IC group were docetaxel, cisplatin plus fluorouracil (DCF) (102 patients, 39.5%), and gemcitabine plus cisplatin (GC) (101 patients, 39.1%). In the AC group, the most commonly administered chemotherapies were cisplatin plus fluorouracil (CF) (103 patients, 70.1%) and DCF (16 patients, 10.9%). In the IC group, dose reduction was applied in 32 patients (12.4%), while 5 patients (1.9%) discontinued treatment early due to toxicity. In the AC group, dose reduction was performed in 10 (6.8%) patients, while treatment was discontinued in 18 (12.2%) patients due to toxicity. The treatments received by the patients are shown in Table 3.

The most commonly observed side effects during treatment were neutropenia (22.9%), anaemia (12.4%), thrombocytopenia (9.3%), and nausea and vomiting (9.3%) in the IC group. In the AC group, the most common side effects were fatigue (11.5%), neutropenia (8.8%), and nausea and vomiting (7.5%). In the IC group, grade 1–2 side effects were observed in 60.5% patients, while grade 3–4 side effects were observed in 12% patients. In the AC group, 29.9% patients had grade 1–2 side effects, while 29.9% patients had grade 3–4 side effects. Treatment-related side effects of the patients are shown in Table 4.

**Figure 1:**
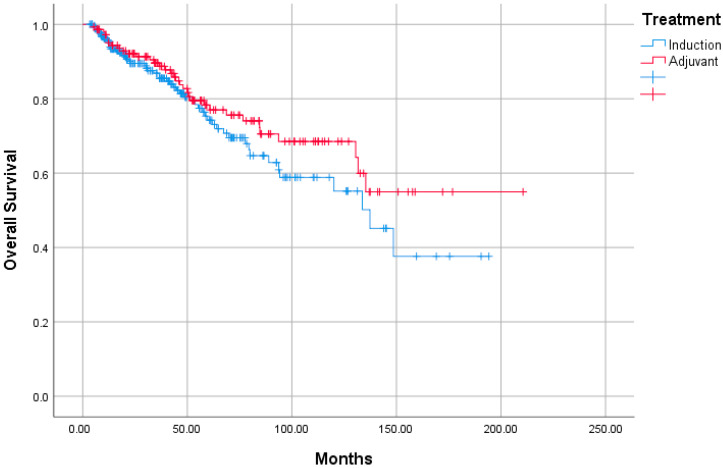
Overall survival rates of induction and adjuvant groups.

**Figure 2:**
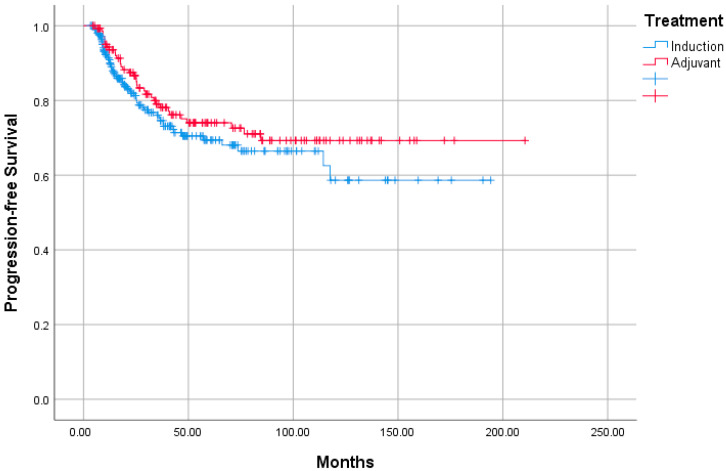
Progression-free survival rates of induction and adjuvant groups.

When OS analysis was performed in terms of age, gender, T and N stages, pathological features, and treatments received, no effect of any variable on prognosis was observed. Similarly, when PFS analysis was performed with the same variables, no factor affecting the prognosis was observed (Table 5). The median estimated survival was 137.3 months (95% confident interval [CI]: 111.4–163.3) in the IC-treated group as a result of OS analysis, while in the AC group, the Kaplan–Meier statistical method could not reach the estimated survival, and no statistical difference was observed between both groups (*p* = 0.209, hazard ratio [HR]: 0.745 [95% CI: 0.484–1.147]) (Figure 1). When PFS analysis was performed, the median estimated survival was not reached by the Kaplan–Meier method in either the IC or the AC group (Figure 2). No statistically significant difference was observed between two groups as a result of PFS analysis (*p* = 0.248, HR:0.797 [95% CI: 0.525–1.209]).

During the follow-up of the patients, 94 (23.2%) patients progressed and 88 (21.7%) patients died. Of the 94 patients with recurrence, 33 (35.1%) had only local recurrence, 33 (35.1%) had local recurrence and distant metastasis, and 28 (29.8%) had only distant metastasis. In the IC group, 58 (22.5%) patients developed recurrence and 54 (20.9%) of these patients died. In the AC group, 36 (24.5%) patients had recurrence and 34 (23.1%) of these patients died. GC (17 patients, 18.1%) and DCF (13 patients, 13.8%) were the most common treatments received after the patients progressed. In total, 13 (13.8%) patients could not receive additional treatment due to their general condition. It was observed that only 9 (9.6%) of the patients could receive second-line treatment after progression.

## 4. Discussion

In this retrospective study, patients diagnosed with locally advanced NPC and treated with IC or AC in combination with CRT were compared. No difference in OS or PFS was observed between IC-CRT and CRT-AC. More grade 3–4 side effects were observed in the CRT-AC group, and early discontinuation of treatment due to toxicity was more common in this group.

Unlike other head and neck cancers, NPC is a tumour for which RT is traditionally the primary treatment because its anatomical structure is not suitable for surgery and it is an RT-sensitive tumour. While RT alone is effective in the treatment of early-stage disease, CRT has been shown to provide better survival results than RT in locally advanced disease [15,16]. There is conflicting information in the literature about the survival outcomes and toxicity profiles of adding AC or IC to CRT. Although some studies have shown that survival results are better with CRT given together with AC or IC, some studies have shown that adding AC or IC only increases toxicity but does not provide any survival advantage [13]. Today, the National Comprehensive Cancer Network (NCCN) states that adding IC or AC to CRT will be beneficial for survival, while IC is more successful in distant disease control [17]. Similarly, the European Society for Medical Oncology (ESMO) recommends IC more than AC because completion rates of IC are higher than AC [5]. In the ESMO guideline, the study by Chen et al. showed that only 60% of patients completed AC treatment, whereas the completion rate of IC treatment was much higher [18].

The recommended chemotherapy during CRT is cisplatin, but it has been reported that carboplatin can be used in cases such as renal failure and hearing loss where the patient is not suitable for cisplatin [19,20]. When the chemotherapies used in IC are analysed in the literature, it has been shown that treatment options such as GC, DCF and CF can be preferred [21,22,23]. When AC treatment options were examined, it was reported that GC or platinum and fluorouracil combinations could be used [24,25]. Most of the patients who participated in our study received cisplatin treatment in combination with RT. While the most commonly used chemotherapies in IC were GC and DCF, the most commonly used chemotherapy in adjuvant treatment was CF.

In the meta-analysis conducted by Blanchard et al., 10-year OS was 57% in patients receiving CRT-AC and 43% in patients receiving CRT alone (HR 0.65, 95% CI 0.56–0.76) [16]. On the other hand, Chen et al. showed that CRT-AC treatment did not contribute to OS, locoregional recurrence-free survival (LRFS) or distant metastasis-free survival (DMFS) compared to CRT, but in addition, the group receiving AC had more toxicity [26].

Zhang et al. conducted a 480-patient randomised controlled phase 3 study and found that 3-year OS was 94.4% in patients with nasopharyngeal cancer who received IC in combination with CRT and 3-year OS was 90.3% in patients who received CRT alone [27]. In the same study, 3-year relapse-free survival (RFS) was 85.3% in the group receiving IC-CRT and 76.5% in the group receiving CRT alone. Majidova et al. showed that PFS and disease control rate (DCR) were higher in patients receiving IC-CRT than in patients receiving CRT alone in a retrospective study of 142 patients in which real life data were shown [28]. In contrast, Yu et al. In a meta-analysis of 2626 patients, no OS difference was found in patients receiving IC-CRT compared to patients receiving CRT alone, and only DMFS of the IC-CRT group was better than that of the CRT group [29]. In a study conducted by Tan et al. with 172 patients, 3-year OS was found to be 94.3% in patients receiving IC-CRT and 92.3% in patients receiving CRT alone and no statistically significant difference was observed (*p* = 0.494) [30]. In the same study, no difference was observed in DFS (*p* = 0.362) or DMFS (*p* = 0.547) between the two groups.

In the literature, there are very few studies comparing AC with IC and the results of these studies are conflicting. In the NPC-0501 study, it was shown that cisplatin and capecitabine treatment as IC contributed to both PFS (HR, 0.72; 95% CI, 0.53–0.99 [*p* = 0.040]) and OS [HR, 0.71; 95% CI, 0.50–1.00 [*p* = 0.048]) compared to cisplatin and fluorouracil treatment as AC [14]. In addition, the toxicity of IC was found to be less than that of AC in this study. On the other hand, Ribassin-Majed et al. reported that CRT-AC gave better OS results than both IC-CRT and CRT in a meta-analysis of 5144 patients [13]. The survival contributions of CRT-AC, CRT, and IC-CRT compared to RT alone were observed as 12%, 8% and 6% at 5 years, respectively. In this study, the toxicity of AC was observed to be higher than IC. In our study, no OS or PFS difference was observed between IC-CRT and CRT-AC treatments. However, more severe toxicities were observed in the group receiving CRT-AC treatment and treatment discontinuation due to toxicity was higher.

There are conflicting results about the effect of EBER positivity on prognosis in nasopharyngeal cancer. Zeng et al. showed that EBER positive patients had a worse prognosis and more recurrences [31]. On the other hand, Sittitrai et al. showed that EBER positivity had no effect on oncological survival and recurrence results [32]. In our study, no significant relationship between EBER positivity and survival was demonstrated.

The limitations of our study are that it is a retrospective study, and the side effects may not have been recorded regularly since the screening was performed from file records. Some pathological data such as EBER and P16 were missing in some clinics. Since the design of our study was retrospective, the treatment of the patients was decided by the tumour boards in the different centres and there were variations between the treatments. Chemotherapy protocols and, albeit rarely, chemotherapy doses used in these treatment modalities showed differences in various centres. Due to the retrospective design, toxicity reporting may have varied across centres. Since the study was conducted in centres in Turkey, treatment responses and side effects may not be generalised to other countries. The biggest impacts of our study are that it is multicentric and it included patients from many regions of Turkey.

## 5. Conclusions

In this retrospective study comparing AC with IC in patients with locally advanced NPC receiving CRT, no difference in OS or PFS was observed between the two treatments. More grade 3–4 side effects were observed in the AC-treated group than in the IC-treated group, and the rate of early discontinuation of treatment due to toxicity was higher in the AC group. More large-scale, prospective studies should be conducted in this subject.

## Figures and Tables

**Table 1:** Baseline characteristics of the patients.

Variables	n (%)
Age, years, median	49.0 (18.2–91.5)
<50 years	215 (53.1%)
≥50 years	190 (46.9%)
Sex	
Male	298 (73.6%)
Female	107 (26.4%)
ECOG	
0	236 (58.3%)
1	163 (40.2%)
2	6 (1.5%)
T stage	
I	70 (17.3%)
II	162 (40%)
III	103 (25.4%)
IV	70 (17.3%)
N stage	
0	15 (3.7%)
I	83 (20.5%)
II	263 (64.9%)
III	44 (10.9%)
Pathological differentiation	
Differentiated	45 (11.1%)
Undifferentiated	311 (76.8%)
No data	49 (12.1%)
Pathological keratinisation	
Keratinised	53 (13.1%)
Nonkeratinised	308 (76.0%)
No data	44 (10.9%)
EBER	
Positive	148 (36.5%)
Negative	75 (18.5%)
No data	182 (44.9%)
P16 protein	
Positive	11 (2.7%)
Negative	92 (22.7%)
No data	302 (74.6%)

EBER: Epstein–Barr virus (EBV)-encoded small RNAs; ECOG: Eastern Cooperative Oncology Group.

**Table 2:** The features of IC and AC groups.

Variables	IC (n = 258)	AC (n = 147)	*p* Value
Age, years, median			0.756
<50 years	135 (52.3%)	80 (54.4%)	
≥50 years	123 (47.7%)	67 (45.6%)	
Sex			0.907
Male	189 (73.3%)	109 (74.1%)	
Female	69 (26.7%)	38 (25.9%)	
T stage			0.251
I–II	142 (55.0%)	90 (61.2%)	
III–IV	116 (45.0%)	57 (32.9%)	
N stage			0.147
0–I	56 (21.7%)	42 (28.6%)	
II–III	202 (78.3%)	105 (71.4%)	
Pathological differentiation			0.067
Differentiated	35 (15.0%)	10 (8.1%)	
Undifferentiated	198 (85.0%)	113 (91.9%)	
Pathological keratinisation			0.161
Keratinised	30 (12.7%)	23 (18.4%)	
Nonkeratinised	206 (87.3%)	102 (81.6%)	
EBER			0.033
Positive	104 (62.3%)	44 (78.6%)	
Negative	63 (37.7%)	12 (21.4%)	
P16 protein			0.999
Positive	9 (11.3%)	2 (8.7%)	
Negative	71 (88.7%)	21 (91.3%)	
Chemotheraphy during CRT			0.999
Carboplatin	14 (5.5%)	8 (5.3%)	
Cisplatin	239 (94.5%)	144 (94.7%)	

AC: Ajuvant chemotherapy; CRT: Chemoradiotherapy; EBER: Epstein–Barr virus (EBV)-encoded small RNAs; IC: Induction chemotheraphy.

**Table 3:** Treatments received of IC and AC groups and completing rates.

Chemotherapy	IC (n = 258)	AC (n = 147)
Cisplatin and fluorouracil	20 (7.8%)	103 (70.1%)
Docetaxel and cisplatin	23 (8.9%)	2 (1.4%)
Docetaxel, cisplatin, and fluorouracil	102 (39.5%)	16 (10.9%)
Docetaxel, carboplatin, and fluorouracil	1 (0.4%)	-
Gemcitabine and cisplatin	101 (39.1%)	13 (8.8%)
Capecitabine	-	3 (2.1%)
Carboplatin and fluorouracil	-	7 (4.8%)
Gemcitabine and carboplatin	3 (1.2%)	3 (2.0%)
Carboplatin and paclitaxel	8 (3.1%)	-
**Treatment results**		
Completed	253 (98.1%)	129 (87.8%)
Dose reduction due to toxicity	32 (12.4%)	10 (6.8%)
Early withdrawal due to toxicity	5 (1.9%)	18 (12.2%)

**Table 4:** Treatment related adverse events.

Adverse Event	IC (n = 258)		AC (n = 147)	
	Grade 1–2	Grade 3–4	Grade 1–2	Grade 3–4
Anaemia	31 (12.0%)	1 (0.4%)	5 (3.4%)	4 (2.7%)
Neutropenia	48 (18.6%)	11 (4.3%)	3 (2.0%)	10 (6.8%)
Thrombocytopenia	22 (8.5%)	2 (0.8%)	2 (1.4%)	4 (2.7%)
Nausea, vomiting	22 (8.5%)	2 (0.8%)	7 (4.8%)	4 (2.7%)
Diarrhoea	6 (2.3%)	2 (0.8%)	2 (1.4%)	-
Fatigue, asthenia	5 (1.9%)	2 (0.8%)	9 (6.1%)	8 (5.4%)
Neuropathy	-	-	2 (1.4%)	-
Mucositis, stomatitis	8 (3.1%)	4 (1.6%)	6 (4.1%)	2 (1.4%)
Rash	2 (0.8%)	-	-	-
Hearing loss	2 (0.8%)	2 (0.8%)	2 (1.4%)	-
Arrhythmia	-	-	-	4 (2.7%)
Elevated liver function test	5 (1.9%)	-	-	-
Alopecia	1 (0.4%)	-	-	-
Febrile neutropenia	-	4 (1.6%)	-	6 (4.1%)
Acute kidney injury	4 (1.6%)	1 (0.1%)	6 (4.1%)	2 (1.4%)
Total	156 (60.5%)	31 (12.0%)	44 (29.9%)	44 (29.9%)

**Table 5:** Prognostic factors of overall survival and progression-free survival in patients.

	*p* Value	
Variables	OS	PFS
Age, <50 years vs. ≥50 years	0.073	0.614
Sex, male vs. female	0.107	0.158
T stage, I–II vs. III–IV	0.744	0.570
N stage, 0–I vs. II–III	0.380	0.947
Pathological differentiation, differentiated vs. undifferentiated	0.129	0.347
Pathological keratinisation, keratinised vs. nonkeratinised	0.805	0.665
EBER, positive vs. negative	0.179	0.469
P16 protein, positive vs. negative	0.149	0.345
Chemotheraphy during CRT, carboplatin vs. cisplatin	0.600	0.801
Treatment, IC vs. AC	0.209	0.248

AC: Adjuvant chemotherapy; CRT: Chemoradiotherapy; EBER: Epstein–Barr virus (EBV)-encoded small RNAs; IC: Induction chemotherapy.

## Data Availability

The datasets generated and/or analysed during the current study are not publicly available due to ethical reasons but are available from the corresponding author on reasonable request. The corresponding author, SS, has to be contacted in case of any queries or requirement of data.

## References

[1] Chua M.L.K., Wee J.T.S., Hui E.P., Chan A.T.C. (2016). Nasopharyngeal carcinoma. Lancet.

[2] Sung H., Ferlay J., Siegel R.L., Laversanne M., Soerjomataram I., Jemal A., Bray F. (2021). Global Cancer Statistics 2020: GLOBOCAN Estimates of Incidence and Mortality Worldwide for 36 Cancers in 185 Countries. CA Cancer J. Clin..

[3] Juarez-Vignon Whaley J.J., Afkhami M., Onyshchenko M., Massarelli E., Sampath S., Amini A., Bell D., Villaflor V.M. (2023). Recurrent/Metastatic Nasopharyngeal Carcinoma Treatment from Present to Future: Where Are We and Where Are We Heading?. Curr. Treat Options Oncol..

[4] Cantù G. (2023). Nasopharyngeal carcinoma. A “different” head and neck tumour. Part B: Treatment, prognostic factors, and outcomes. Acta Otorhinolaryngol. Ital..

[5] Bossi P., Chan A.T., Licitra L., Trama A., Orlandi E., Hui E.P., Halámková J., Mattheis S., Baujat B., Hardillo J. (2021). Nasopharyngeal carcinoma: ESMO-EURACAN Clinical Practice Guidelines for diagnosis, treatment and follow-up(†). Ann. Oncol..

[6] Al-Sarraf M., LeBlanc M., Giri P.G., Fu K.K., Cooper J., Vuong T., Forastiere A.A., Adams G., Sakr W.A., Schuller D.E. (1998). Chemoradiotherapy versus radiotherapy in patients with advanced nasopharyngeal cancer: Phase III randomized Intergroup study 0099. J. Clin. Oncol..

[7] Chen Q.Y., Wen Y.F., Guo L., Liu H., Huang P.Y., Mo H.Y., Chen Q.-Y., Luo D.-H., Huang P.-Y., Cao K.-J. (2011). Concurrent chemoradiotherapy vs radiotherapy alone in stage II nasopharyngeal carcinoma: Phase III randomized trial. J. Natl. Cancer Inst..

[8] Chen Y., Sun Y., Liang S.B., Zong J.F., Li W.F., Chen M., Chen L., Mao Y.-P., Tang L.-L., Guo Y. (2013). Progress report of a randomized trial comparing long-term survival and late toxicity of concurrent chemoradiotherapy with adjuvant chemotherapy versus radiotherapy alone in patients with stage III to IVB nasopharyngeal carcinoma from endemic regions of China. Cancer.

[9] Petit C., Lee A., Ma J., Lacas B., Ng W.T., Chan A.T.C., Hong R.-L., Chen M.-Y., Chen L., Li W.-F. (2023). Role of chemotherapy in patients with nasopharynx carcinoma treated with radiotherapy (MAC-NPC): An updated individual patient data network meta-analysis. Lancet Oncol..

[10] Li W.F., Chen N.Y., Zhang N., Hu G.Q., Xie F.Y., Sun Y., Chen X.-Z., Li J.-G., Zhu X.-D., Hu C.-S. (2019). Concurrent chemoradiotherapy with/without induction chemotherapy in locoregionally advanced nasopharyngeal carcinoma: Long-term results of phase 3 randomized controlled trial. Int. J. Cancer.

[11] Sun Y., Li W.F., Chen N.Y., Zhang N., Hu G.Q., Xie F.Y., Sun Y., Chen X.-Z., Li J.-G., Zhu X.-D. (2016). Induction chemotherapy plus concurrent chemoradiotherapy versus concurrent chemoradiotherapy alone in locoregionally advanced nasopharyngeal carcinoma: A phase 3, multicentre, randomised controlled trial. Lancet Oncol..

[12] Lv X., Cao X., Xia W.X., Liu K.Y., Qiang M.Y., Guo L., Qian C.-N., Cao K.-J., Mo H.-Y., Li X.-M. (2021). Induction chemotherapy with lobaplatin and fluorouracil versus cisplatin and fluorouracil followed by chemoradiotherapy in patients with stage III–IVB nasopharyngeal carcinoma: An open-label, non-inferiority, randomised, controlled, phase 3 trial. Lancet Oncol..

[13] Ribassin-Majed L., Marguet S., Lee A.W.M., Ng W.T., Ma J., Chan A.T.C., Huang P.-Y., Zhu G., Chua D.T.T., Chen Y. (2017). What Is the Best Treatment of Locally Advanced Nasopharyngeal Carcinoma? An Individual Patient Data Network Meta-Analysis. J. Clin. Oncol..

[14] Lee A.W.M., Ngan R.K.C., Ng W.T., Tung S.Y., Cheng A.A.C., Kwong D.L.W., Lu T.-X., Chan A.T.C., Sze H.C.K., Yiu H.H.Y. (2020). NPC-0501 trial on the value of changing chemoradiotherapy sequence, replacing 5-fluorouracil with capecitabine, and altering fractionation for patients with advanced nasopharyngeal carcinoma. Cancer.

[15] Au K.H., Ngan R.K.C., Ng A.W.Y., Poon D.M.C., Ng W.T., Yuen K.T., Lee V.H.F., Tung S.Y., Chan A.T.C., Sze H.C.K. (2018). Treatment outcomes of nasopharyngeal carcinoma in modern era after intensity modulated radiotherapy (IMRT) in Hong Kong: A report of 3328 patients (HKNPCSG 1301 study). Oral. Oncol..

[16] Blanchard P., Lee A., Marguet S., Leclercq J., Ng W.T., Ma J., Chan A.T.C., Huang P.-Y., Benhamou E., Zhu G. (2015). Chemotherapy and radiotherapy in nasopharyngeal carcinoma: An update of the MAC-NPC meta-analysis. Lancet Oncol..

[17] Caudell J.J., Gillison M.L., Maghami E., Spencer S., Pfister D.G., Adkins D., Birkeland A.C., Brizel D.M., Busse P.M., Cmelak A.J. (2022). NCCN Guidelines^®^ Insights: Head and Neck Cancers, Version 1.2022: Featured Updates to the NCCN Guidelines. J. Natl. Compr. Cancer Netw..

[18] Chen L., Hu C.S., Chen X.Z., Hu G.Q., Cheng Z.B., Sun Y., Li W.-X., Chen Y.-Y., Xie F.-Y., Liang S.-B. (2012). Concurrent chemoradiotherapy plus adjuvant chemotherapy versus concurrent chemoradiotherapy alone in patients with locoregionally advanced nasopharyngeal carcinoma: A phase 3 multicentre randomised controlled trial. Lancet Oncol..

[19] Lee A.W., Tung S.Y., Ngan R.K., Chappell R., Chua D.T., Lu T.X., Siu L., Tan T., Chan L.K., Ng W.T. (2011). Factors contributing to the efficacy of concurrent-adjuvant chemotherapy for locoregionally advanced nasopharyngeal carcinoma: Combined analyses of NPC-9901 and NPC-9902 Trials. Eur. J. Cancer.

[20] Chitapanarux I., Lorvidhaya V., Kamnerdsupaphon P., Sumitsawan Y., Tharavichitkul E., Sukthomya V., Ford J. (2007). Chemoradiation comparing cisplatin versus carboplatin in locally advanced nasopharyngeal cancer: Randomised, non-inferiority, open trial. Eur. J. Cancer.

[21] Zhang Y., Chen L., Hu G.Q., Zhang N., Zhu X.D., Yang K.Y., Jin F., Shi M., Chen Y.-P., Hu W.-H. (2022). Final Overall Survival Analysis of Gemcitabine and Cisplatin Induction Chemotherapy in Nasopharyngeal Carcinoma: A Multicenter, Randomized Phase III Trial. J. Clin. Oncol..

[22] Lorch J.H., Goloubeva O., Haddad R.I., Cullen K., Sarlis N., Tishler R., Tan M., Fasciano J., Sammartino D.E., Posner M.R. (2011). Induction chemotherapy with cisplatin and fluorouracil alone or in combination with docetaxel in locally advanced squamous-cell cancer of the head and neck: Long-term results of the TAX 324 randomised phase 3 trial. Lancet Oncol..

[23] Cao S.M., Yang Q., Guo L., Mai H.Q., Mo H.Y., Cao K.J., Qian C.-N., Zhao C., Xiang Y.-Q., Zhang X.-P. (2017). Neoadjuvant chemotherapy followed by concurrent chemoradiotherapy versus concurrent chemoradiotherapy alone in locoregionally advanced nasopharyngeal carcinoma: A phase III multicentre randomised controlled trial. Eur. J. Cancer.

[24] Liu L.T., Liu H., Huang Y., Yang J.H., Xie S.Y., Li Y.Y., Guo S.S., Qi B., Li X.Y., Chen D.P. (2023). Concurrent chemoradiotherapy followed by adjuvant cisplatin-gemcitabine versus cisplatin-fluorouracil chemotherapy for N2–3 nasopharyngeal carcinoma: A multicentre, open-label, randomised, controlled, phase 3 trial. Lancet Oncol..

[25] Lee A.W., Tung S.Y., Chan A.T., Chappell R., Fu Y.T., Lu T.X., Tang T., Chua D.T.T., O’Sullivan B., Tung R. (2011). A randomized trial on addition of concurrent-adjuvant chemotherapy and/or accelerated fractionation for locally-advanced nasopharyngeal carcinoma. Radiother. Oncol..

[26] Chen Y.P., Wang Z.X., Chen L., Liu X., Tang L.L., Mao Y.P., Li W.F., Lin A.H., Sun Y., Ma J. (2015). A Bayesian network meta-analysis comparing concurrent chemoradiotherapy followed by adjuvant chemotherapy, concurrent chemoradiotherapy alone and radiotherapy alone in patients with locoregionally advanced nasopharyngeal carcinoma. Ann. Oncol..

[27] Zhang Y., Chen L., Hu G.Q., Zhang N., Zhu X.D., Yang K.Y., Jin F., Ma J. (2019). Gemcitabine and Cisplatin Induction Chemotherapy in Nasopharyngeal Carcinoma. N. Engl. J. Med..

[28] Majidova N., Sarı M., Kahvecioglu F.A., Ozcan E., Akdag M.O., Dogan A., Yıldırım S., Sonusen S.D., Yunusov E., Yasar A. (2024). Clinicopathologic Features and Efficacy of Induction Chemotherapy in Nasopharyngeal Carcinoma: Real-World Experience. Oncol. Res. Treat..

[29] Yu H., Gu D., He X., Gao X., Bian X. (2016). The role of induction and adjuvant chemotherapy in combination with concurrent chemoradiotherapy for nasopharyngeal cancer: A Bayesian network meta-analysis of published randomized controlled trials. OncoTargets Ther..

[30] Tan T., Lim W.T., Fong K.W., Cheah S.L., Soong Y.L., Ang M.K., Ng Q.S., Tan D., Ong W.S., Tan S.H. (2015). Concurrent chemo-radiation with or without induction gemcitabine, Carboplatin, and Paclitaxel: A randomized, phase 2/3 trial in locally advanced nasopharyngeal carcinoma. Int. J. Radiat. Oncol. Biol. Phys..

[31] Zeng M.C., Jia Q.J., Xu J., Chen J.J., Qin K., Xu L.M. (2023). The whole-blood Epstein-Barr virus DNA can serve as a valuable molecular marker for diagnosis and prognosis prediction of nasopharyngeal carcinoma. Am. J. Cancer Res..

[32] Sittitrai P., Ruenmarkkaew D., Chitapanarux I., Muangwong P., Kangsadarnwiroon K., Benjawongsatien R., Srivanitchapoom C., Donchalermpak S., Asakij T. (2024). Head and Neck Cancer of Unknown Primary: A Multicenter Retrospective Cohort study in Northern Thailand, an Endemic Nasopharyngeal Cancer Area. Asian Pac. J. Cancer Prev..

